# Culture creates genetic structure in the Caucasus: Autosomal, mitochondrial, and Y-chromosomal variation in Daghestan

**DOI:** 10.1186/1471-2156-9-47

**Published:** 2008-07-17

**Authors:** Elizabeth E Marchani, W Scott Watkins, Kazima Bulayeva, Henry C Harpending, Lynn B Jorde

**Affiliations:** 1 Department of Anthropology, University of Utah, Salt Lake City, UT 84112, USA; 2 Eccles Institute of Human Genetics, University of Utah, Salt Lake City, UT 84112, USA; 3 N.I. Vavilov Institute of General Genetics, Russian Academy of Sciences, Moscow 117991, Russia

## Abstract

**Background:**

Near the junction of three major continents, the Caucasus region has been an important thoroughfare for human migration. While the Caucasus Mountains have diverted human traffic to the few lowland regions that provide a gateway from north to south between the Caspian and Black Seas, highland populations have been isolated by their remote geographic location and their practice of patrilocal endogamy. We investigate how these cultural and historical differences between highland and lowland populations have affected patterns of genetic diversity. We test 1) whether the highland practice of patrilocal endogamy has generated sex-specific population relationships, and 2) whether the history of migration and military conquest associated with the lowland populations has left Central Asian genes in the Caucasus, by comparing genetic diversity and pairwise population relationships between Daghestani populations and reference populations throughout Europe and Asia for autosomal, mitochondrial, and Y-chromosomal markers.

**Results:**

We found that the highland Daghestani populations had contrasting histories for the mitochondrial DNA and Y-chromosome data sets. Y-chromosomal haplogroup diversity was reduced among highland Daghestani populations when compared to other populations and to highland Daghestani mitochondrial DNA haplogroup diversity. Lowland Daghestani populations showed Turkish and Central Asian affinities for both mitochondrial and Y-chromosomal data sets. Autosomal population histories are strongly correlated to the pattern observed for the mitochondrial DNA data set, while the correlation between the mitochondrial DNA and Y-chromosome distance matrices was weak and not significant.

**Conclusion:**

The reduced Y-chromosomal diversity exhibited by highland Daghestani populations is consistent with genetic drift caused by patrilocal endogamy. Mitochondrial and Y-chromosomal phylogeographic comparisons indicate a common Near Eastern origin of highland populations. Lowland Daghestani populations show varying influence from Near Eastern and Central Asian populations.

## Background

The populations of the Caucasus region have complex histories of isolation and gene flow. The region as a whole has served as a gateway between continents, with waves of human migration leaving rich cultural and linguistic diversity in their wake [1,2]. The Caucasus Mountains have shaped the routes of migrating populations and military invasions, diverting these travellers away from the remote highlands and into the more easily accessible lowlands. Differences between highland and lowland populations are exaggerated by the marriage practices of highland populations: wives move to the home of their husbands, while husbands remain in the land of their forefathers for generations [3,4].

We have identified five populations from Daghestan that have been influenced by both physical and cultural barriers to gene flow. Three are highland isolates, while two lowland populations represent admixed groups influenced by Turkic and Mongolian migrants. We investigate whether the geographic barrier of altitude, the cultural barrier of patrilocal endogamy, or the introduction of migrants from a great distance have left detectable patterns in the genetic diversity of these populations. Specifically, we ask 1) whether geographic isolation and patrilocal endogamy have caused more genetic drift in highland than lowland populations, and 2) whether lowland populations show evidence of admixture from Turkic and Mongolian migrants.

We test these hypotheses by comparing mitochondrial DNA (mtDNA) and Y-chromosome (non-recombining, NRY) haplogroup frequencies among Daghestani, Near Eastern, Central Asian, Central/Northern European, and East Asian populations, as well as autosomal variation in 100 polymorphic *Alu *insertions among Daghestani, Central/Northern European, and East Asian populations. We compare measures of haplogroup diversity and pairwise distance for the mtDNA and NRY markers, then compare these to genetic distances from the autosomal data, looking for evidence of genetic drift and shared origins. Our results demonstrate that the cultural practice of patrilocality and historic population movements have shaped genetic variation in these Caucasus populations.

## Methods

### Populations

**Figure 1:**
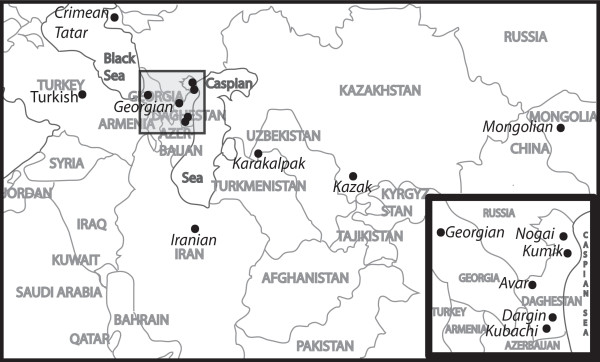
**Map of Population Sample Locations**. This simplified and stylized map illustrates the location associated with each population sampled. Central/Northern European and East Asian samples are as described in the Methods section.

The Avar, Dargin, and Kubachi populations sampled in our study live in the highlands of Daghestan (Figure 1). They each speak different languages belonging to the Northeast Caucasian language family. The Avars and Dargins have traditionally been agriculturalists and pastoralists, while the Kubachi have specialized in jewelry making [5]. These highland populations are isolated due to their remote location, their linguistic variation, and their practice of strict patrilocal endogamy [3,4]. This marriage practice controls the inheritance of property and restricts male gene flow [3]. These populations are thought to be indigenous to the region but, like other native peoples of the Caucasus region, their exact origins are unclear [6]. Previous genetic studies have revealed that populations within the Caucasus region do not share the genetic variation believed by some researchers to be a signature of the Neolithic expansion through Europe, leading some to infer that these populations are remnants of a more ancient Eurasian population [3,7,8]. Others suggest that the Caucasus region is instead inhabited by a collection of peoples who represent those who have travelled through or invaded the region in the historic past [4,9,10].

The Nogai and Kumiks of the Daghestani lowlands have a history of admixture. These populations speak different languages belonging to the Kipchak division of the Turkic language family and are more exogamous than the highland populations [11,12]. The Kumik population represents a mixture of native peoples of the Caucasus with Turkic migrants from the 4^th ^to the 15^th ^centuries and may be descended from the Kipchaks, an ancient Turkic population [10,12]. The Kumiks currently practice a flexible form of virilocality and frequently exchange mates with other villages. The Nogai are descended from the Nogai khanate of the Mongol empire, established in the 12^th ^century, which arrived in Daghestan in the 13^th ^and 14^th ^centuries [5,11]. Although administrated by Mongolians, this khanate was peopled by many native Caucasian ethnic groups [11,12], suggesting that the Nogai are an admixed population. The Nogai practice dual exogamy, prohibiting marriage within one's kin group or patronymic, a practice that encourages gene flow.

Overall, the five ethnic groups sampled for our study are representative of the genetic variation to be found in Daghestan, as these groups represent approximately one-half of the country's population [3]. DNA samples were obtained following voluntary consent procedures developed and approved by the Daghestan IRB at the Institute of History, Archeology and Ethnology of Daghestan Center, of the Russian Academy of Sciences.

To place the genetic variation observed within Daghestan into a regional perspective, we include mtDNA and NRY data obtained from the literature for several Near Eastern and Central Asian populations in our analyses. Near Eastern populations include Crimean Tatars [2,13], Georgians [2,14], Iranians [2,13], and Turks [14,15]. The Central Asian populations included in this study are Kazak [2,13], Karakalpak [2,13], and Mongolian [2,16].

We are unable to include these Near Eastern and Central Asian population in our analyses of autosomal Alu insertion variation, as comparable data have not been published. We therefore include Central/Northern European and East Asian populations in our study of mtDNA, NRY, and Alu insertion variation. Our Central/Northern European sample includes 15 French and 57 Utahns of Central/Northern European ancestry, while the East Asian population includes 9 Cambodians, 13 Han Chinese, 16 Japanese, 5 Malay, 3 Taiwanese, and 9 Vietnamese individuals. These populations are geographically and genetically more distant from the Daghestani populations than the Near Eastern and Central Asian populations. As such, they act as extreme values in pairwise distance calculations and principal components analyses. These populations place the observed differences between Daghestani and other populations into a broader perspective. Distances between Daghestani populations and the Central/Northern European and East Asian populations serve as a yardstick against which distances within Daghestan, and between Daghestani populations and populations from the Near East and Central Asia may be measured.

### Mitochondrial DNA Haplogroup Variation

We have genotyped 62 Avar, 28 Dargin, 25 Kubachi, 26 Kumik, 33 Nogai, 54 East Asian, and 71 Central/Northern European individuals for the following 24 mtDNA haplogroup-defining single nucleotide polymorphisms (SNPs) using ABI SNaPshot (Applied Biosystems, Foster City, CA) multiplexes designed by WSW [17]: A1811G, T4646C, A4833G, C4883T, G7598A, A7768G, G7805A, A7933G, C8137T, G8994A, G9055A, T9090C, G9266A, A9545G, C10400T, T10436C, C10810T, C10873T, A12308G, A12612G, T12705C, A13263G, T14766C, A14793G. We have refined these data by identifying mtDNA haplogroup defining SNPs [16,18-20] in the HVS-I sequences of our samples. We performed new HVS-I sequencing for our Avar samples, while the sequences for the other Daghestani populations are available in the literature [3]. We were able to define 26 mtDNA haplogroups with these data, with the combinations of SNPs used to define each mtDNA haplogroup in our samples listed in additional file 1: mtDNAdefinitions.xls. MtDNA haplogroup frequencies among Near Eastern and Central Asian populations were obtained from the literature [13,14,16].

### Y-chromosome Haplogroup Variation

In a similar fashion, we have genotyped the males of the above sample (18 Avar, 8 Dargin, 14 Kubachi, 10 Kumik, 16 Nogai, 31 East Asian, and 61 Central/Northern European individuals) for the following 19 NRY haplogroup-defining SNPs using ABI SNaPshot (Applied Biosystems, Foster City, CA) multiplexes designed by WSW [17]: M122, M145, M17, M170, M172, M173, M174, M175, M181, M20, M201, M207, M216, M52, M74, M89, M9, M91, M96. NRY haplogroup frequencies among Near Eastern and Central Asian populations were obtained from the literature [2,15]. We follow the phylogeny and nomenclature established by the Y Chromosome Consortium [21], with the combination of SNPs used to define each haplogroup listed in additional file 2: NRYdefinitions.xls.

### Autosomal Alu Insertion Variation

Genotypes for 100 Alu insertions have been previously published [22] for the Dargin, Kubachi, Kumik, Nogai, Central/Northern European and East Asian individuals genotyped for the mtDNA SNPs.

### Analytical Methods

Haplogroup diversity within Daghestani populations was calculated using Nei's *h *statistic [23]. The pairwise genetic distances between populations were calculated using population pairwise F_ST_. Genetic distances were tested for significant differences from zero with 10,000 permutations in a randomization test (Arlequin 2.0; [24]). Principal components analyses were performed using MATLAB R2007b in order to visualize the genetic relationships between populations. Mantel tests, using 10,000 random permutations in Arlequin 2.0, were performed to assess the correlations among the NRY, mtDNA, and Alu distance matrices.

## Results

### Haplogroup Frequencies and Diversity

Mitochondrial haplogroup frequencies and diversity values are presented in Table 1. The Daghestani populations resemble our samples from Central/Northern Europe and Turkey in their frequencies of common haplogroups HV, T, and U5. The highland Kubachi are an exception, with more than 75% of the sample possessing subtypes of haplogroup U, most commonly observed in Central/Northern Europe and Russia [25]. The lowland Nogai samples resemble the samples from Central and East Asia, as all have appreciable frequencies of haplogroups B, C, and D. The range of haplogroup diversity values within Daghestan lies within the range observed among other populations, with neither highland (Avar, Dargin, Kubachi) nor lowland (Kumik, Nogai) Daghestani populations exhibiting unusually high or low values.

**Table 1:** Mitochondrial DNA Haplogroup Frequencies and Diversity.

	**Avar**	**Dargin**	**Kubachi**	**Kumik**	**Nogai**	**Crimean Tatar^1^**	**Georgian^2^**	**Iranian^1^**	**Turkish^2^**	**Karakalpak^1^**	**Kazak^1^**	**Mongolian^3^**	**N. Europe**	**East Asian**
**Sample Size**	62	28	25	26	33	20	58	20	90	20	20	97	71	54
**A**	0.00	0.04	0.00	0.00	0.00	0.00	0.00	0.00	0.02	0.00	0.00	0.08	0.00	0.00
**B**	0.00	0.00	0.00	0.00	0.06	0.00	0.00	0.00	0.00	0.00	0.00	0.06	0.00	0.09
**C**	0.00	0.00	0.08	0.00	0.09	0.00	0.00	0.10	0.00	0.00	0.05	0.08	0.00	0.02
**D**	0.00	0.00	0.00	0.00	0.15	0.00	0.00	0.05	0.01	0.20	0.10	0.30	0.00	0.22
**E**	0.00	0.00	0.00	0.00	0.00	0.00	0.00	0.00	0.00	0.00	0.00	0.00	0.00	0.00
**F**	0.00	0.00	0.00	0.00	0.00	0.00	0.00	0.00	0.00	0.05	0.00	0.13	0.00	0.15
**G**	0.00	0.07	0.00	0.04	0.03	0.00	0.00	0.00	0.01	0.05	0.20	0.10	0.00	0.02
**HV**	0.20	0.43	0.12	0.46	0.27	0.20	0.22	0.15	0.36	0.25	0.20	0.03	0.55	0.00
**J**	0.06	0.04	0.00	0.04	0.00	0.10	0.09	0.10	0.09	0.05	0.05	0.00	0.11	0.00
**K**	0.08	0.07	0.04	0.04	0.09	0.10	0.09	0.10	0.06	0.00	0.00	0.00	0.10	0.00
**L2**	0.00	0.00	0.00	0.00	0.00	0.00	0.00	0.00	0.00	0.00	0.00	0.00	0.00	0.00
**M**	0.00	0.00	0.00	0.00	0.06	0.00	0.02	0.00	0.00	0.10	0.05	0.08	0.00	0.39
**N**	0.06	0.04	0.00	0.00	0.00	0.10	0.09	0.25	0.06	0.00	0.00	0.04	0.00	0.06
**R**	0.00	0.00	0.00	0.00	0.00	0.00	0.05	0.10	0.02	0.10	0.05	0.01	0.03	0.04
**T**	0.10	0.07	0.00	0.04	0.06	0.30	0.14	0.05	0.11	0.05	0.05	0.01	0.10	0.00
**U**	0.00	0.00	0.00	0.00	0.00	0.00	0.00	0.00	0.02	0.00	0.00	0.00	0.00	0.00
**U1**	0.03	0.00	0.48	0.12	0.00	0.05	0.05	0.05	0.07	0.10	0.05	0.00	0.00	0.00
**U2**	0.00	0.00	0.24	0.00	0.03	0.00	0.03	0.00	0.02	0.00	0.00	0.00	0.00	0.00
**U3**	0.00	0.07	0.00	0.00	0.00	0.10	0.03	0.00	0.02	0.00	0.00	0.00	0.00	0.00
**U4**	0.05	0.11	0.00	0.00	0.03	0.05	0.03	0.00	0.01	0.00	0.00	0.01	0.01	0.00
**U5**	0.05	0.07	0.04	0.08	0.03	0.00	0.02	0.05	0.06	0.00	0.10	0.03	0.07	0.00
**U6**	0.00	0.00	0.00	0.00	0.00	0.00	0.00	0.00	0.00	0.00	0.00	0.00	0.00	0.00
**U7**	0.00	0.00	0.00	0.00	0.00	0.00	0.00	0.00	0.02	0.00	0.00	0.00	0.00	0.00
**W**	0.23	0.00	0.00	0.15	0.00	0.00	0.05	0.00	0.01	0.00	0.00	0.00	0.03	0.00
**X**	0.15	0.00	0.00	0.04	0.00	0.00	0.09	0.00	0.03	0.00	0.00	0.00	0.00	0.00
**Y**	0.00	0.00	0.00	0.00	0.00	0.00	0.00	0.00	0.00	0.00	0.05	0.00	0.00	0.00
**Z**	0.00	0.00	0.00	0.00	0.09	0.00	0.00	0.00	0.00	0.05	0.05	0.02	0.00	0.04
**Nei's *h***	0.84	0.75	0.66	0.71	0.84	0.78	0.87	0.82	0.83	0.81	0.84	0.85	0.65	0.75

Haplogroups as defined in Additional File 1. Superscripts refer to the reference number of the source data.

References: 1. Comas et al. (2004); 2. Quintana-Murci et al. (2004); 3. Yao et al. (2004)

NRY haplogroup frequencies and diversity values are presented in Table 2. The highland Avar, Dargin, and Kubachi populations exhibit high frequencies of haplogroup F (including haplogroup G, see Discussion), which is also common in many Near Eastern populations. The lowland Nogai and Kumik samples exhibit a mixture of European, Near Eastern, and Central Asian haplogroups. The lowland Kumik and Nogai populations have NRY haplogroup diversity values as high as those observed in the Near East and Central Asia, while the highland Daghestani populations have lower NRY haplogroup diversity, less than or equal to that observed in our Central/Northern European sample. The highland Dargins are exceptional, with a Nei's *h *value of zero, as all males sampled belong to NRY haplogroup F.

**Table 2:** Y-Chromosome Haplogroup Frequencies and Diversity.

	**Avar**	**Dargin**	**Kubachi**	**Kumik**	**Nogai**	**Crimean Tatar^1^**	**Georgian^2^**	**Iranian^1^**	**Turkish^2^**	**Karakalpak^1^**	**Kazak^1^**	**Mongolian^3^**	**C/N Europe**	**East Asian**
**Sample Size**	18	8	12	10	16	22	67	52	523	44	54	24	61	31
A	0.00	0.00	0.00	0.00	0.00	0.00	0.00	0.00	0.00	0.00	0.00	0.00	0.00	0.00
C	0.00	0.00	0.00	0.00	0.13	0.09	0.00	0.00	0.01	0.22	0.66	0.59	0.00	0.03
D	0.00	0.00	0.00	0.00	0.06	0.05	0.04	0.19	0.00	0.00	0.00	0.00	0.00	0.03
E	0.00	0.00	0.00	0.00	0.00	0.00	0.00	0.00	0.11	0.00	0.02	0.04	0.00	0.00
F (F*xH,I,J2)	0.61	1.00	0.92	0.30	0.06	0.18	0.50	0.40	0.20	0.09	0.02	0.08	0.02	0.00
H	0.00	0.00	0.00	0.20	0.00	0.00	0.00	0.00	0.01	0.00	0.00	0.00	0.00	0.00
I	0.00	0.00	0.00	0.00	0.00	0.05	0.02	0.00	0.05	0.00	0.00	0.00	0.36	0.00
J2	0.33	0.00	0.00	0.10	0.06	0.14	0.33	0.23	0.24	0.09	0.00	0.00	0.00	0.00
K (K*xL,O)	0.00	0.00	0.00	0.20	0.25	0.00	0.00	0.02	0.06	0.09	0.02	0.13	0.00	0.03
L	0.00	0.00	0.00	0.00	0.00	0.00	0.00	0.04	0.04	0.05	0.00	0.00	0.00	0.00
O (O*xO3)	0.00	0.00	0.00	0.00	0.06	0.05	0.00	0.00	0.00	0.00	0.02	0.04	0.00	0.39
O3	0.00	0.00	0.00	0.00	0.00	0.05	0.00	0.02	0.00	0.11	0.09	0.08	0.00	0.52
P (P*x R1)	0.00	0.00	0.00	0.00	0.00	0.00	0.00	0.06	0.03	0.07	0.07	0.00	0.02	0.00
R1*	0.06	0.00	0.08	0.20	0.25	0.09	0.06	0.02	0.16	0.09	0.06	0.00	0.56	0.00
R1a1	0.00	0.00	0.00	0.00	0.13	0.32	0.06	0.02	0.07	0.18	0.04	0.04	0.05	0.00
**Nei's *h***	0.48	0.00	0.14	0.70	0.78	0.79	0.63	0.72	0.85	0.84	0.53	0.60	0.55	0.56

Haplogroups as defined in Additional File 2. Superscripts refer to the reference number of the source data.

References: 1. Wells et al.(2001); 2. Cinnioglu et al.(2004); The symbol "x" means "excluding the following subtypes."

Haplogroups G, H, J (J*xJ2) have been genotyped for our Daghestani, Central/Northern European, and East Asian samples, though they are unavailable for the reference populations. Because of this, haplogroup F includes individuals who may belong to haplogroups G or J (xJ2).

### Population Pairwise Distances

Genetic distances based on mtDNA haplogroup frequencies are shown in the upper triangle of Table 3, while the NRY pairwise distances are shown in the lower triangle. The NRY population pairwise distances show no differentiation between the highland Daghestani populations, while the Avar and Dargin highland populations are significantly different from the highland Kubachi with respect to the mtDNA. This suggests that either the history of Daghestani Y chromosomes differs from that of the mtDNA and autosomal loci, or that the males genotyped for the NRY polymorphisms are not sufficiently representative of the population at large. The Kumik and Turkish populations have F_ST _values of zero, while the Nogai have low genetic distances to the Near Eastern and Central Asian populations, for both the mtDNA and NRY data sets.

**Table 3:** Mitochondrial DNA and Y-chromosome Pairwise F_ST _Values.

	Avar	Dargin	Kubachi	Kumik	Nogai	Crimean Tatar	Georgian	Iranian	Turkish	Karakalpak	Kazak	Mongolian	C/N European	East Asian
Avar	*	0.07	**0.19**	0.04	**0.12**	0.06	0.02	0.05	**0.06**	**0.12**	**0.12**	**0.27**	**0.16**	**0.33**
Dargin	0.14	*	**0.20**	0.00	0.08	0.01	0.02	0.05	0.00	0.10	0.11	**0.30**	0.02	**0.38**
Kubachi	0.14	0.00	*	**0.20**	**0.25**	**0.20**	**0.18**	**0.24**	**0.19**	**0.29**	**0.27**	**0.43**	**0.34**	**0.51**
Kumik	0.11	0.13	0.06	*	0.09	0.05	0.02	0.06	0.00	0.09	0.11	**0.30**	0.04	**0.39**
Nogai	**0.25**	0.24	0.20	0.02	*	0.10	**0.09**	0.02	**0.10**	0.00	0.00	**0.08**	**0.17**	0.12
Crimean Tatar	0.19	0.17	0.15	0.04	0.00	*	0.00	0.03	0.00	**0.12**	0.13	**0.31**	0.08	**0.40**
Georgian	0.00	0.07	0.07	0.06	**0.20**	0.16	*	0.01	0.00	**0.10**	0.11	**0.27**	**0.08**	**0.34**
Iranian	0.03	0.05	0.06	0.05	**0.16**	0.15	0.03	*	0.04	0.02	0.02	**0.15**	0.13	**0.23**
Turkish	0.05	0.08	0.06	0.00	0.05	0.06	0.04	**0.06**	*	0.10	**0.13**	**0.30**	0.03	**0.36**
Karakalpak	0.18	0.15	0.13	0.03	0.00	0.00	**0.16**	**0.14**	0.06	*	0.00	0.07	**0.19**	0.11
Kazak	**0.36**	0.31	**0.31**	0.28	**0.23**	**0.26**	**0.33**	**0.27**	**0.24**	**0.16**	*	0.05	**0.22**	0.09
Mongolian	**0.34**	0.29	**0.30**	0.25	0.21	**0.22**	**0.30**	**0.22**	**0.21**	0.13	0.00	*	**0.39**	0.00
C/N European	**0.39**	**0.38**	0.33	0.18	0.09	0.12	**0.34**	**0.33**	**0.17**	**0.14**	**0.42**	**0.43**	*	**0.46**
East Asian	**0.63**	**0.67**	**0.65**	**0.52**	**0.42**	**0.41**	**0.53**	**0.47**	**0.37**	**0.32**	**0.51**	**0.52**	**0.53**	*

Mitochondrial DNA results in upper triangle, Y-chromosome results in lower triangle.

Bold values are significantly different from zero with Bonferroni correction, p < 0.05

The autosomal pairwise genetic distances calculated from the 100 Alu loci (Table 4) are similar to those observed for the mtDNA data but not the NRY data. Mantel tests of correlation between the mtDNA, NRY, and autosomal distance matrices (Table 5) demonstrate a strong and significant correlation between the mtDNA and Alu distances, a weaker correlation between the NRY and Alu distances, and no significant correlation between the NRY and mtDNA distance matrices.

**Table 4:** Autosomal Alu Insertion Pairwise F_ST _Values.

	Dargin	Kubachi	Kumik	Nogai	C/N European	East Asian
Dargin	*					
Kubachi	**0.02**	*				
Kumik	0.00	0.03	*			
Nogai	0.02	**0.04**	0.02	*		
C/N European	0.02	**0.03**	0.01	**0.03**	*	
East Asian	**0.07**	**0.09**	**0.06**	0.02	**0.08**	*

Bold values are significantly different from zero with Bonferroni correction, p < 0.05.

**Table 5:** Mantel Correlation Test Results

**Comparison**	**R**	**p**
Alu insertions vs. Mitochondrial DNA Haplogroups	0.94	<0.005
Alu insertions vs. Y-chromosome Haplogroups	0.78	<0.05
Mitochondrial DNA vs. Y-chromosome Haplogroups	0.31	0.09

### Principal Components Analysis

**Figure 2:**
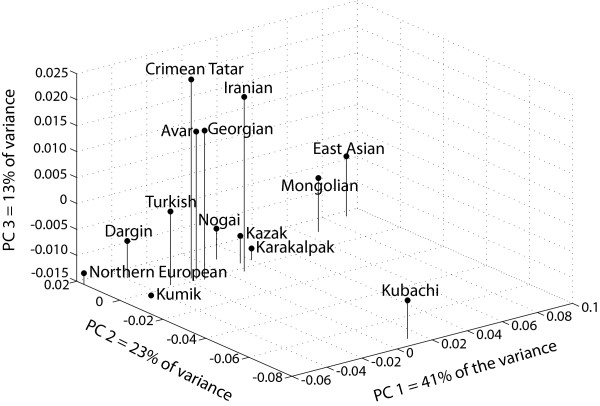
Principal Components Analysis of Mitochondrial DNA Haplogroup Data.

Figure 2 presents the principal components analysis results for the mtDNA haplogroup frequency data. The first principal component, explaining 41% of the variance, identifies the Central/Northern European and East Asian populations as the extremes. Populations fall along the first principal components axis in a rough west-to-east gradient, with the exception of the Daghestani populations. The highland Kubachi are isolated by the second principal component, explaining 23% of the variance. This is likely due to genetic drift leading to the observed high frequencies of mtDNA haplogroups U1 (48%) and U2 (24%) within the Kubachi population.

The third principal component, explaining 13% of the variance, identifies the lowland Kumik and Crimean Tatar populations as the extremes. This third principal component almost divides the Near Eastern populations from the Central Asian populations. Highland Avars cluster with the Near Eastern reference populations, while the highland Dargins and lowland Kumiks are intermediate between the Central/Northern European and Turkish populations. The lowland Nogai lie between the Near Eastern and Central Asian populations.

**Figure 3:**
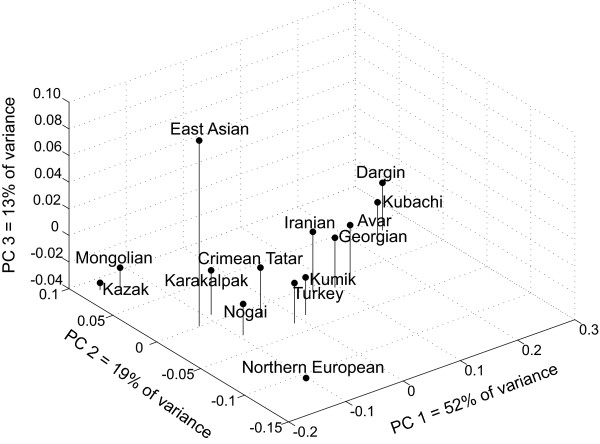
Principal Components Analysis of Y-chromosome Haplogroup Data.

Figure 3 presents the results of the NRY principal components analysis. The first principal component, explaining 52% of the variance, separates the highland Daghestani populations from the Asian populations. The second principal component separates the Central/Northern European population from the Central Asian populations, explaining 19% of the variance. The third principal component isolates the East Asian population, explaining 13% of the variance. The lowland Kumiks cluster with the Near Eastern populations, while the Nogai lie between the Central Asian and Near Eastern populations.

**Figure 4:**
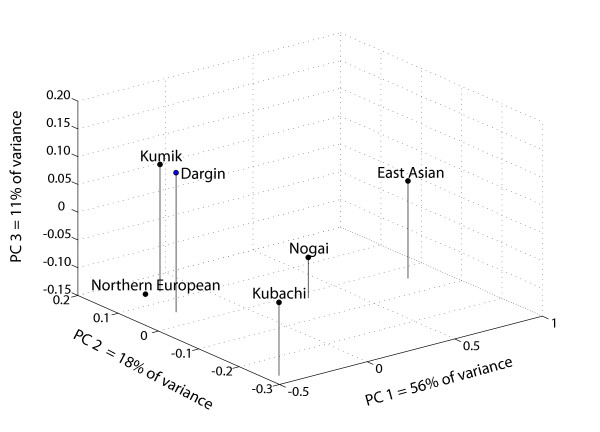
**Principal Components Analysis of Autosomal Alu Insertion Data**. Near Eastern and Central Asian reference populations are excluded from this analysis as comparable data do not exist.

Figure 4 portrays a principal components analysis of the autosomal Alu data. Although the Near Eastern and Central Asian populations are missing, the Alu results are strikingly similar to the mtDNA results of Figure 2. The first principal component separates the Central/Northern European population from the East Asian population, explaining 56% of the variance. The second principal component isolates the Kubachi, explaining 18% of the variance. The highland Dargins and lowland Kumiks cluster with the Central/Northern Europeans, while the highland Kubachi are isolated by the second principal component. The Nogai are at an intermediate position between the Central/Northern European and East Asian populations for each of the three axes.

## Discussion

Cultural restrictions on marriage practices have proved to be powerful barriers to gene flow, as evidenced by the historical caste system in India [26] and patrilocal marriage practices throughout the world [27-29]. Because highland Daghestani populations practice patrilocal endogamy, we would expect that they would exhibit reduced genetic diversity and larger genetic distances when compared to other populations with respect to the NRY but not mtDNA. Our observations are consistent with these predictions. We see a reduction of genetic diversity in the NRY among highland populations compared to that in other populations and to their own mtDNA diversity. This pattern of reduction is not observed for the lowland Daghestani populations.

We see no significant correlation between the mtDNA and NRY distance matrices. Y chromosomes of highland Daghestanis appear to have undergone genetic drift independent of the population history of mitochondrial and autosomal loci, suggesting that restrictions on marriage, and not geographic isolation, are the causal agents for NRY drift among highland populations. This is consistent with previous research [30], which found that the mountain range had not acted as a significant barrier to gene flow between populations located north and south of the Caucasus Mountains.

The highland Kubachi form an endogamous isolate of nearly 3,000 individuals [5], restricting both male and female gene flow. Our results indicate genome-wide signs of genetic drift for this population. For each set of markers, the Kubachi population has high pairwise distance values to lowland Daghestani populations as well as to reference populations. These patterns of genetic isolation are reflected in the mtDNA and autosomal principal components analyses, where the Kubachi are isolated from other populations by the second principal component.

Patrilocal endogamy should have resulted in each highland population drifting to different extremes, as the stochastic process of genetic drift occurs independently in each population. Instead, we see that all Daghestani highland populations share high frequencies of NRY haplogroup F in contrast to their neighbours. When we break haplogroup F down to include haplogroups G and J, greater detail emerges. The highland Avar, Dargin, and Kubachi exhibit high frequencies of haplogroup J (0.56, 1.00, and 0.67, respectively), though haplogroup F is still present among the Kubachi (0.25), and haplogroup G is observed among the Avar (0.06), Kumik (0.10), and Nogai (0.06) populations. The NRY haplogroup frequencies among highland Daghestani populations are consistent with a shared common ancestral population dominated by NRY haplogroup J and other haplogroups observed among Near Eastern and Indian populations [31-33].

Phylogeographic analysis of the mtDNA haplogroups found in highland Daghestani populations also suggests Near Eastern and Caucasus-specific origins [31,32,34,35]. Among the highland Daghestani populations, we observe polymorphic frequencies of Near Eastern mitochondrial haplogroups U1 and W and Caucasian haplogroups U3, U4, and X. An extreme case is observed among the Kubachi, who maintain high frequencies of mitochondrial haplogroups U1 (48%) and U2 (24%), though these haplogroups are generally rare in other populations. Together, the mitochondrial and NRY haplogroup histories suggest that the highland Daghestani populations possess genetic variation that has not been substantially influenced by recent migrants.

Genetic variation among the lowland Kumik and Nogai populations reveals a diverse heritage influenced to varying degrees by Turkic and Mongolian migrants. Both the Kumiks and Nogai possess high levels of mtDNA and NRY haplogroup diversity, consistent with multiethnic origins. Kumiks show genetic affiliation with Near Eastern populations for all measures of mtDNA and NRY genetic variation, with F_ST _distances to the Turkish population equalling zero for both analyses. This is consistent with historical evidence of a Turkic origin of the Kumik population.

The Nogai population is defined by its relationship to the administration of the Mongol Empire in the Caucasus, when Mongolians ruled over the native peoples of the Caucasus region [11,12]. We might expect that this historical event would have mainly introduced Central Asian males to the native populations of the Caucasus, and would have subsequently introduced more Central Asian Y chromosomes than mtDNA variants to the Caucasian gene pool [36]. If instead, the Nogai represent a population of both males and females that migrated to the Caucasus region, we would expect the mtDNA and NRY histories to be similar to each other. In fact, this is the case for the Kalmyks, a Russian population who migrated from Mongolia approximately 300 years ago [37].

The Nogai do show clear evidence of an Asian heritage. Approximately half of the mitochondrial and NRY haplogroups observed among the Nogai are of Asian origin (mtDNA: M, C, Z, D, G; NRY: C, D, O, O3) [34,35]. They are also placed between the Near Eastern and Central Asian populations for both mtDNA and NRY principal components analyses. Autosomal Alu analyses places the Nogai at an intermediate position between the East Asian and other Daghestani populations, and are consistent with the pattern observed in the mtDNA analyses. The relationships between the Nogai and Central Asian and Near Eastern populations are consistent across marker types, showing the importance of including Near Eastern and Central Asian populations in our analyses. It is quite possible that our small (*N *= 16) sample of Nogai males is not representative of the full spectrum of NRY genetic variation in the population. Asymmetric gene flow and differing effective population sizes across loci could also cause differing population pairwise distances to Central/Northern European and East Asian reference populations for these markers.

## Conclusion

This study describes the effects of culture, geography, and gene flow on genetic diversity among Daghestani populations. Highland populations show reduced Y-chromosome diversity, but autosomal and mtDNA variation is not reduced, reflecting the effects of a patrilocal mating system. Mitochondrial and Y-chromosomal phylogeographic inferences suggest a Near Eastern or Caucasus-region origin of the highland populations. Our results, including haplogroup sharing, genetic diversity, and patterns of population pairwise distance lead us to confirm that the lowland Kumik and Nogai populations have been influenced by gene flow from local and migrant Central Asian populations, as suggested by history. Overall, our results demonstrate that population history, geographic isolation, and patrilocality have all left detectable signatures on the genetic landscape of the Caucasus.

## Authors' contributions

EEM designed the study, genotyped the Daghestani individuals, performed the analysis, and drafted the manuscript. WSW designed the multiplex technology used to genotype the mitochondrial and Y-chromosome haplogroups and genotyped the Central/Northern European and East Asian individuals. KB collected the DNA samples from Daghestani populations. HCH and LBJ participated in study design, supervision, and revision of the manuscript.

## Supplementary Material

Additional file 1 **Mitochondrial DNA Haplogroup Definitions**. Describes how Daghestani, East Asian, and Central/Northern European samples were assigned to haplogroups. Click here for file

Additional file 2 **Y-chromosome Haplogroup Definitions**. Describes how Daghestani, East Asian, and Central/Northern European samples were assigned to haplogroups. Click here for file
